# Corrosion Prevention of Aluminum Nanoparticles by a Polyurethane Coating

**DOI:** 10.3390/ma7064710

**Published:** 2014-06-19

**Authors:** Toshiyasu Nishimura, Vedarajan Raman

**Affiliations:** Institute for Materials Science (NIMS), 1-2-1 Sengen, Tsukuba, Ibaraki 305-0047, Japan; E-Mail: raman@jaist.ac.jp

**Keywords:** corrosion, nanoparticle, aluminum, reaction, coating, polymer

## Abstract

In order to prevent corrosion, aluminum nanoparticles were coated with a polyurethane polymer. The coverage of the polyurethane polymer was controlled from 0 to 100%, which changed the corrosion rate of the nanoparticles quantitatively. The surface of the polymer coating was investigated by Transmission Electron Microscopy (TEM) and Atomic Force Microscopy (AFM), and the corrosion resistance of the nanoparticles was estimated by a wet/dry corrosion test on a Pt plate with a NaCl solution. From a TEM with EDAX analysis, the 10 mass% polymer coated Al particles in the synthesis were almost 100% covered on the surface by a polymer film of 10 nm thick. On the other hand, the 3 mass% polymer coated Al was almost 40% covered by a film. In the AFM, the potential around the Al particles had a relatively low value with the polymer coating, which indicated that the conductivity of the Al was isolated from the Pt plate by the polymer. Both the corrosion and H_2_ evolution reaction rates were quantitatively reduced by the mass% of polymer coating. In the case of the 10 mass% coated sample, there was no corrosion of Al nanoparticles. This fact suggested that the electrochemical reaction was suppressed by the polymer coating. Moreover, the reaction rate of Al nanoparticles was suppressed in proportion to the coverage percentage of the coating. Thus, to conclude, it was found that the corrosion rate of Al nanoparticles could be quantitatively suppressed by the coverage percentage of the polymer coating.

## 1. Introduction

In general, nanoscience is a well developed area with respect to synthesis, characterization, exploration, and exploitation of nanostructured materials. These materials are characterized as having at least one side of length in the nanometer range. Thus, the surface area of nanomaterials is increased remarkably compared with bulk materials, enabling them to have dramatically increased surface reaction rates. From the point of view of high surface area materials, the size effect of nanomaterials has been investigated in the fields of catalysis [1,2,3,4,5], sensors [6,7,8] and batteries [9,10].

Although the reaction rate can be increased by making the particle nano-sized, however then, the deterioration of the nano metal can be caused by corrosion under the atmosphere. In general, the nanoparticles need to be stored as raw materials for many production processes. Then, corrosion problems usually occur under high humidity conditions. It is a problem that corrosion reduces the quality of nanoparticles by forming oxides.

Moreover, in the case of coastal areas, the air borne salt particles coming from the sea, cause severe corrosion conditions. Besides, the film thickness of the coating should be extremely thin because each nanoparticle needs to be coated separately without bonding to one another in the synthesis. Although it is necessary to prevent the corrosion of metal nanoparticles, there are few papers on this problem.

In this paper, we have attempted to prevent the corrosion of aluminum (Al) nanoparticles using a polyurethane polymer coating. The coverage percentage of the polymer coating was varied from 0 to 100% to investigate the corrosion behavior of the nanometals quantitatively. The coverage film was investigated by nano-level investigation using TEM and AFM. Then, the corrosion reaction was examined for the polymer coated nano Al on a Pt plate by a wet and dry test using a NaCl solution [11]. Using these results, the relationship between the coverage percentage of polymer and the corrosion rate of the nanometal was discussed. Finally the corrosion prevention mechanism of the nanometal was investigated by changing the coverage percentage of the polymer film quantitatively.

## 2. Experimental Procedure

## 2.1. Creation of Polymer Coated Al Nanoparticles

The Al nanoparticles were prepared using an atomizing method. Melted Al was atomized and sieved as particles 300–600 nanometers (nm) in diameter. The Al surface oxide was completely removed in 0.5 M H_2_SO_4_ solution, thus markedly improving the surface activity. Moreover, this process is very important for conducting the synthesis of the polymer only on the metal nanoparticles.

**Figure 1:**
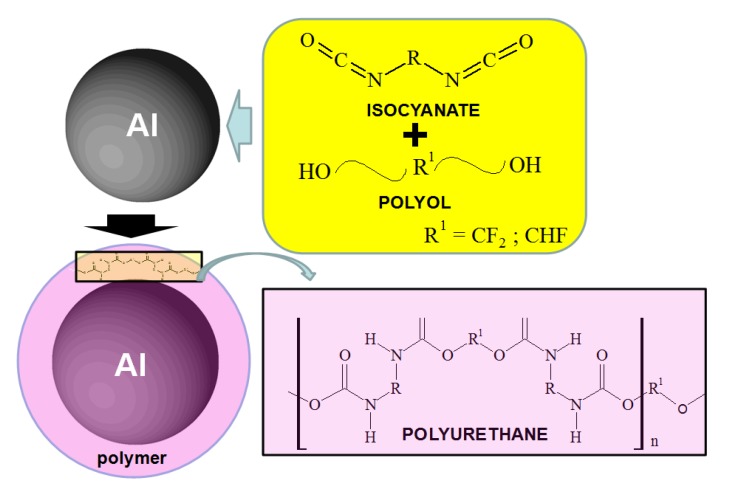
Schematic diagram of the synthesis reaction for the polymer on Al nanoparticles.

**Figure 2:**
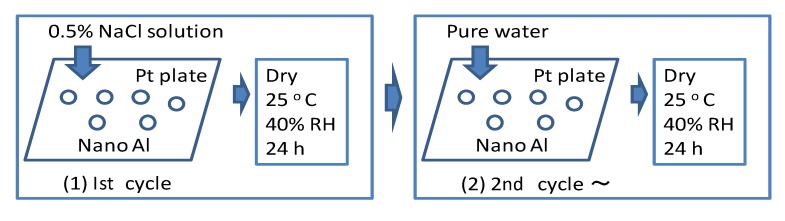
Schematic diagram of the wet and dry corrosion test.

The polyurethane polymer was synthesized on the Al nanoparticle by the chemical reaction of isocyanate and polyol as shown in Figure 1 and Figure 2. Fluorine (F) was found as either CF_2_ or CHF in the polyol. The urethane polymer was dissolved in isopropyl alcohol with the Al nanoparticles, and sufficiently agitated to wet the surface of the nanoparticles. After the isopropyl alcohol was completely evaporated under vacuum, the polyurethane polymer was hardened at atmospheric pressure. Then, the polymer was completely consumed to make a thin film on the nanoparticles. The mass% ratios of the polymer to Al in the synthesis were 0%, 0.1%, 0.4%, 1.0%, 3.0% and 10.0%, in order to change the coverage percentage of polymer film on the surface of the nanoparticle. As is explained below, the coverage percentage was only able to be controlled by the mass% ratio of polymer in the synthesis.

## 2.2. Surface Analysis of the Polymer Film on Nano Al

As for TEM, Energy Dispersive X-ray Spectroscopy (EDXS) was used to estimate the coverage percentage of the polymer on the Al nanoparticles by measuring the amount of F in the polymer as a marker. In order to investigate the chemical state of the polymer film in more detail, Electron Energy Loss Spectroscopy (EELS) spectra were observed by TEM [12]. The accelerated voltage was 200 kV, and the half width of value of zero loss was 1 eV. Moreover the nano level investigation on the sample was conducted by Atomic Force Microscopy (AFM) and Kelvin Force Microscopy (KFM) measurements. The machine for AFM/KFM is a SPA400 (Seiko Instruments SII with SPI 3800N probe station, Chiba, Japan). First, the Al nanoparticles were dispersed in methanol, ultrasonicated for 5 min, placed on a polished Pt plate, and then AFM and KFM investigations were begun. In the AFM measurement, the surface morphology and potential distribution (KFM) measurements were conducted simultaneously with the tapping mode using gold coated cantilevers with a spring constant of 2.1 N/m.

## 2.3. Corrosion Analysis

Wet and dry corrosion tests (Figure 2) were conducted for 20 days in a 0.5 mass% NaCl solution for 20 days (cycles) to estimate the corrosion behavior of the polymer coated Al nanoparticles. Only for the first cycle of testing, a 0.5 mass% NaCl solution was poured over the Al nanoparticles on the platinum (Pt) plate in a chamber kept at 40% relative humidity (RH) and 25 °C. Under this condition, the sample was dried for several hours. Then, from the 2nd cycle of the test, pure water was poured over the samples under the same conditions every 24 h. As the NaCl remains on the Pt plate, it has corrosive conditions after the 2nd cycle of the test. During the corrosion test the samples are not removed.

There is no corrosion of Al nanoparticle without contact with the Pt plate. However, the anodic dissolution of Al occurs by galvanic contact with Pt which has a large cathodic area. Finally, in order to evaluate the corrosion rate, the sizes of the Al nanoparticles were measured after a wet and dry corrosion test. The size of the Al nanoparticles was normalized for 20 samples by the supposition that the average of the initial diameter of particles was 100%.

After the corrosion test, the corrosion behavior of the nanoparticles was observed by SEM, AFM and KFM. It was found that the corrosion rate was suppressed in proportion to the coverage percentage of the polymer coating. Finally, a corrosion prevention mechanism for the nanometal was demonstrated by changing the surface coverage percentage of polymer coating quantitatively.

Besides this, the effect of polymer coating mass% on the corrosion of Al nanoparticles in an alkali solution was examined. The volume of H_2_ evolution for 70 mg of Al nanoparticles was measured in a 0.5 M NaOH solution. H_2_ gas was collected using a glass apparatus in the solution. In order to reduce problems in the experiment, the test time was shortened.

## 3. Results and Discussion

## 3.1. Surface Analysis of the Polymer Coated Al Nanoparticles

**Figure 3:**
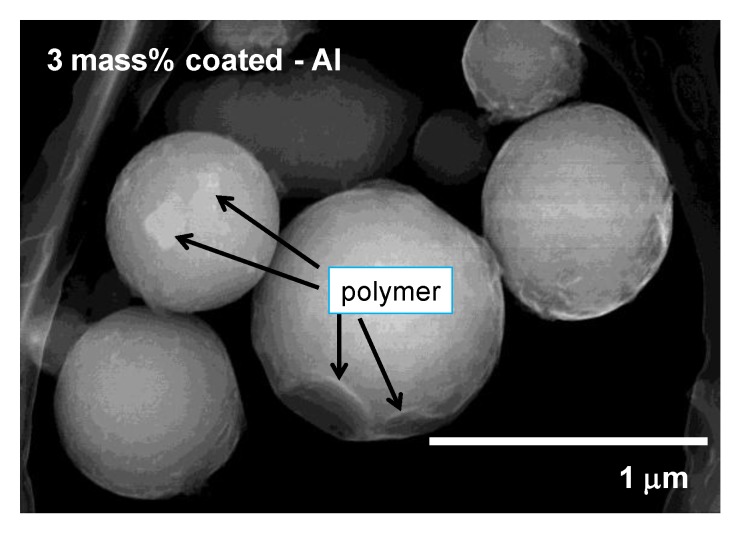
The secondary electron image of the 3 mass% polymer coated Al nanoparticles by Scanning Electron Microscopy (SEM).

**Figure 4:**
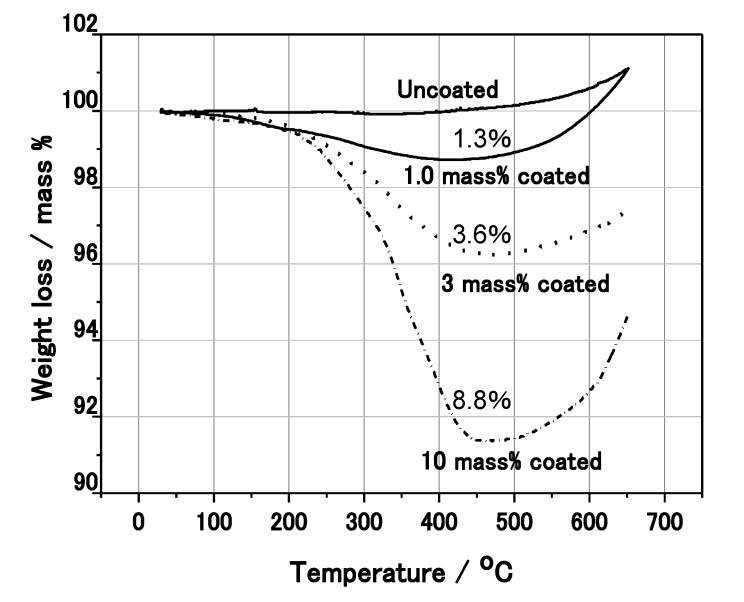
Thermo Gravimetric Analysis (TGA) of the coated Al nanoparticles.

Figure 3 shows the secondary electron image of the 3 mass% polymer coated Al nanoparticles by SEM. Here, 3 mass% means that the polymer is coated as a weight ratio of 3 mass% against the weight of Al. It was found that the particle diameter was 300–600 nm, and the particles were partially covered by the polymer. In order to investigate the actual mass% of polymer to Al nanoparticles, thermo-gravimetric analysis (TGA) was conducted under a N_2_ atmosphere (Figure 4). As the temperature was increased above 200 °C, the weight of the nanoparticle decreased due to the melt of the polymer. The weight loss of the 1, 3 and 10 mass% coated Al nanoparticles was 1.3, 3.6 and 8.8 mass%, respectively, as measured with TGA. As the sensitivity of TGA is not high, the weight loss from the Al nanoparticles is almost the same as the mass% initially used in the synthesis. Thus, almost all of the polymer synthesized remains on the surface of the nanoparticles. It is possible to control the mass% of actual polymer coating using the mass% of polymer to Al in the synthesis.

**Figure 5:**
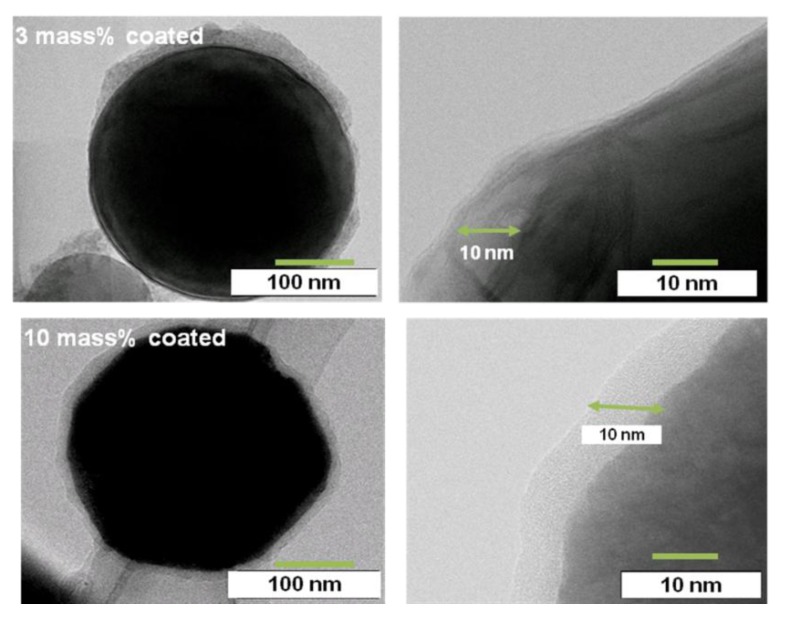
Transmission Electron Microscopy (TEM) images of the 3 mass% and 10 mass% coated Al nanoparticles.

In order to investigate the coating state of the polyurethane polymer in more detail, TEM images were made as shown in Figure 5. For the 10 mass% coated sample, a 10 nm thick polymer uniformly coats the entire surface of the Al nanoparticles. Thus the amount of 10 mass% of polymer coating is thought to be enough to cover all of the surface of the Al nanoparticle. On the other hand, the 3 mass% sample is only partially coated. However, the thickness of the polymer is also 10 nm in the areas where the particles are coated. In fact, the polymer thickness was almost 10 nm in all of the polymer samples. Although a thickness of 10 nm is perhaps needed in order to maintain the film, the detailed mechanism is unknown. In this way, as the film thickness is very thin, each nanoparticle is able to be coated separately without bonding to one another in the synthesis. This is very important to coat the nanometal while maintaining its characteristics.

**Figure 6:**
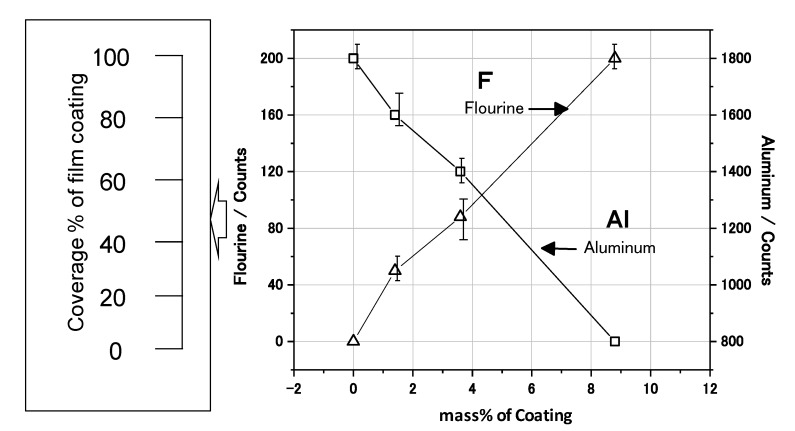
Energy Dispersive X-ray Spectroscopy (EDXS) analysis of F and Al for the Al nanoparticles, and coverage percentage of the film coating calculated by EDXS counts.

The EDXS mappings were measured to estimate the coverage state of the film coating on the Al nanoparticle. The EDXS concentration of fluorine (F) was higher in the case of the 10 mass% coated sample than the 3 mass% coated one. In Figure 6 the EDXS count of F increases linearly as the mass% of the coating increases. Since F is contained in the polymer as CF_2_ or CHF, the EDXS count of F is proportional to the coating coverage percentage. Thus, the coating coverage percentage can be practically estimated from the EDXS F counts. As for the 10 mass% sample, 100% coverage is identified by the TEM in Figure 6, and the count is almost 200. Thus, the coverage percentage for the 3 mass% sample is estimated to be almost 40% from an F count of 90. Similarly, the coverage percentage for the 1 mass% sample is estimated to be almost 25% from an F count of 50. In this way, the coverage percentage of film coating is able to be practically estimated by the EDXS counts of F. Thus, it has been demonstrated that the coverage percentage of the developed samples can be controlled from 0 to 100% by changing the mass% of polymer coating in the synthesis. In addition, it has been found that the relationship between the film coverage percentage and the coating mass% can be obtained by TEM analysis.

**Figure 7:**
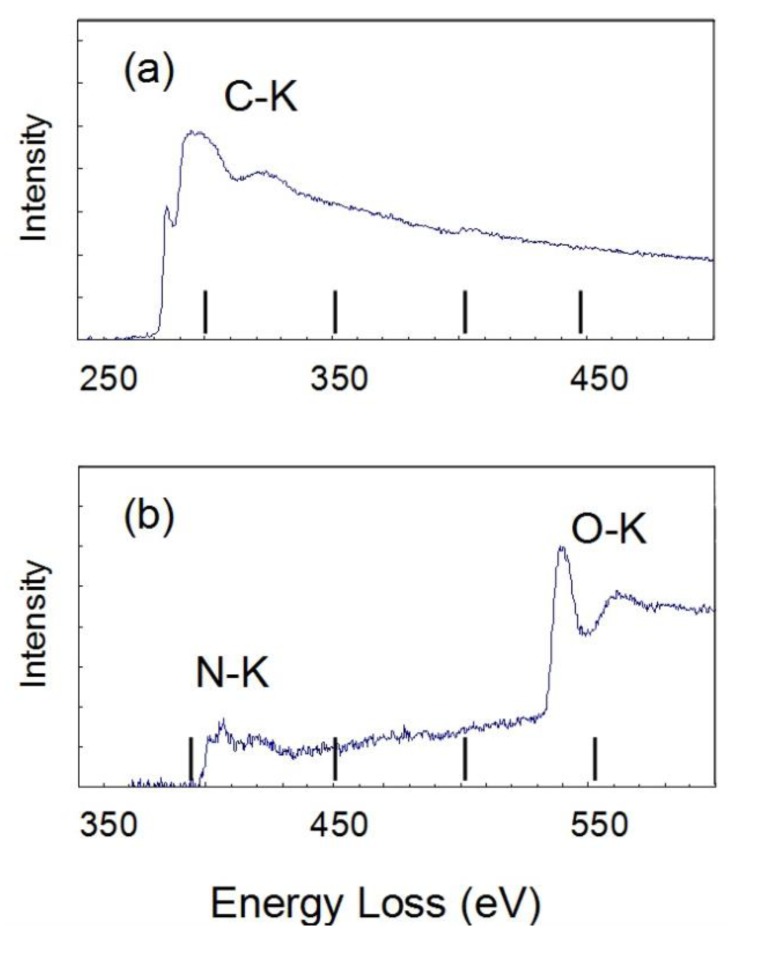
Electron Energy Loss spectroscopy (EELS) spectra of (**a**) C-K and (**b**) N-K, O-K for 10 mass% coated Al.

**Figure 8:**
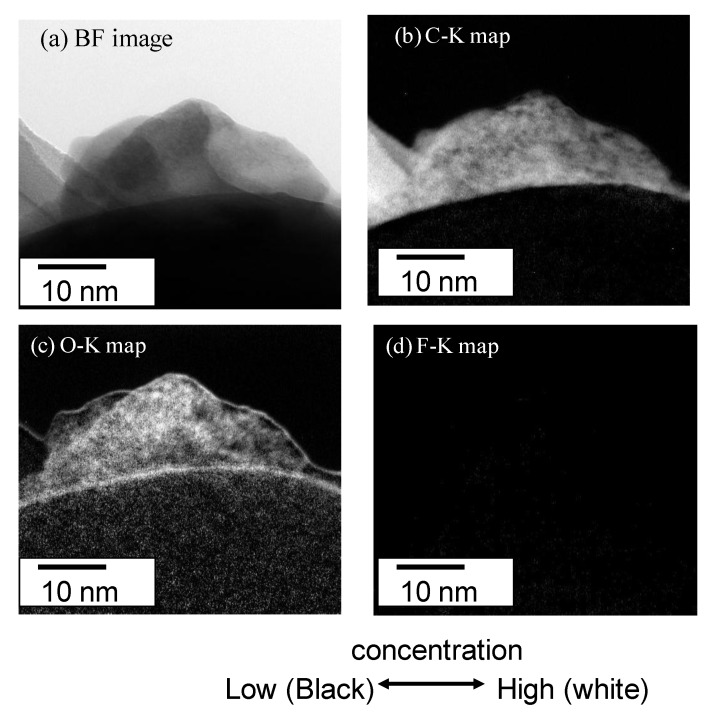
(**a**) Bright field image; and EELS mappings of (**b**) C-K, (**c**) O-K, (**d**) F-K for 10 mass% coated Al.

The Electron Energy Loss spectroscopy (EELS) spectra were obtained for the 10 mass% coated Al to estimate the chemical state of C, N, and O in the polymer film in Figure 7. There are strong peaks for C-K at 284, N-K at 410 and O-K at 535 eV. On the other hand, there is a very weak peak for F-K at 690 eV. The spectrum of C-K shows the typical peak showing the σ-bond. These peaks of C-K, N-K and O-K are thought to show the chemical bond of the polyurethane polymer. The Bright field (BF) image (a) and EELS mappings are shown in (b) C-K, (c) O-K and (d) F-K for the 10 mass% coated Al in Figure 8. The BF image shows a polymer film of almost 10 nm. The high concentration of C and O are shown in the polymer film. However, the concentration of C and O are very low on the particle because the thickness of the Al particle is very high. Thus, the high concentration of C-K and O-K in the film is thought to show the chemical bond of polyurethane polymer. The mapping of F is not detected clearly because the peak strength is weak. The EELS spectra are detected only from the film outside of the particle, which is a very narrow area. On the other hand, EDXS are taken from all over the film on the particle. Thus the sensitivity and the reliability in EDXS for F are much higher than in EELS.

## 3.2. Corrosion of the Polymer-Coated Al Nanoparticles

**Figure 9:**
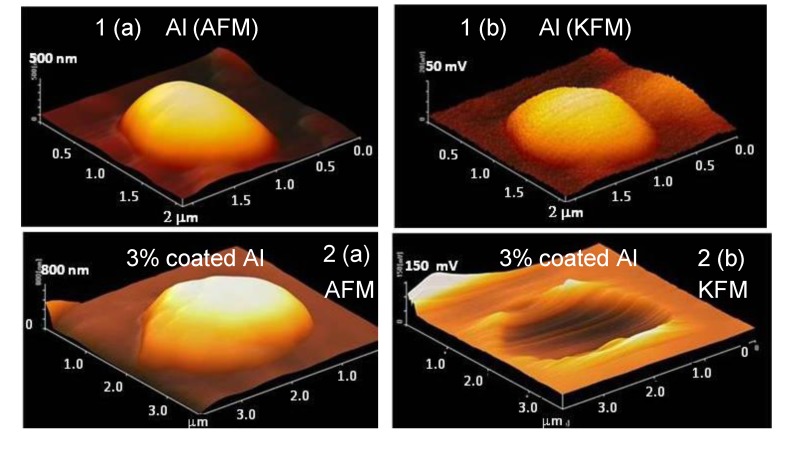
Atomic Force Microscopy (AFM) and Kelvin Force Microscopy (KFM) (the potential distribution) images of Al nanoparticles as 3-dimensional representations. (**a**) AFM images of the nanoparticle of uncoated Al (1) and a 3 mass% polymer coated Al (2); (**b**) KFM images of uncoated Al (1) and a 3 mass% polymer coated Al (2).

AFM and KFM (potential distribution) measurements were carried out using the tapping mode as shown in Figure 9. The KFM image for the uncoated Al particle is represented by a half globe, almost the same as the AFM image. On the other hand, although the AFM images for the 3 mass% coated Al particle is a half globe, the KFM image displays a broken shape. Thus, the potential around the Al particles is thought to be partially isolated from the Pt plate by the polymer coating. This fact suggests that the electrochemical reaction is suppressed by the coverage of the polymer. In other words, it is possible to prevent the corrosion reaction by coverage of the polymer film.

**Figure 10:**
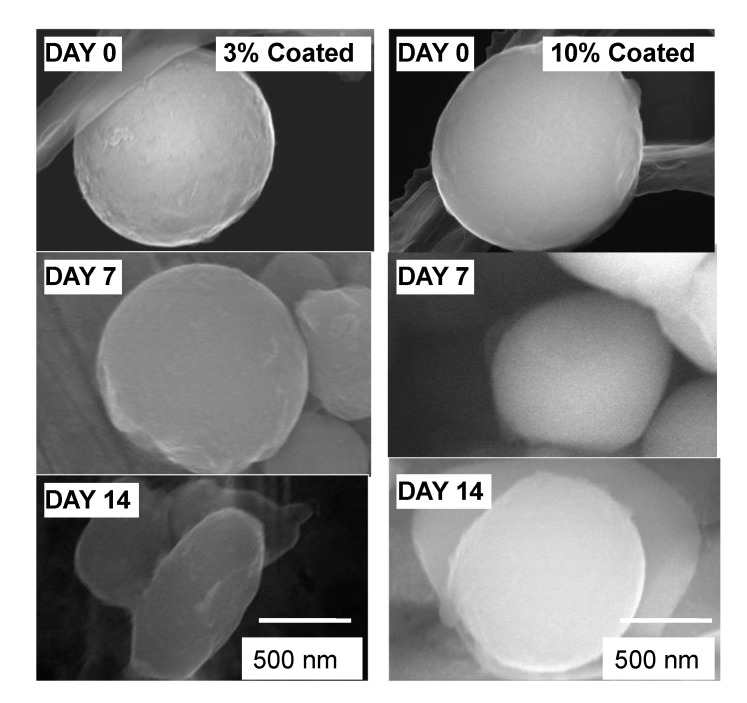
SEM images of Al nanoparticles subjected to a wet and dry corrosion test in Figure 2.

A wet and dry corrosion test using a NaCl solution was conducted. A 0.5 mass% NaCl solution was poured over the Al nanoparticles in a chamber kept at 40% relative humidity (RH) and 25 °C. Figure 10 shows SEM images of the Al nanoparticles taken after the corrosion test at each day. The shape of the 10 mass% polymer coated sample is unchanged after 14 days, showing that it has not been subjected to corrosion. This corrosion prevention is due to complete coverage with polymer which isolates Al particles from the Pt plate. However, the 3 mass% sample is partially corroded at 7 days, and continues to be corroded, yielding a slender-shaped particle after 14 days. Actually, the diameter of the particle is reduced by 20% at the area of the highest corrosion at 7 days. Additionally, it is reduced by 60% at 14 days. In this case, the corrosion is thought to start in an area of bare (no polymer) metal, and the electrochemical reaction continues in that region. Finally the galvanic corrosion between Al particle and the Pt plate is thought to continue for 14 days. In this way, the shape of the Al nanoparticle varies with the polymer coating percentage after the corrosion test.

**Figure 11:**
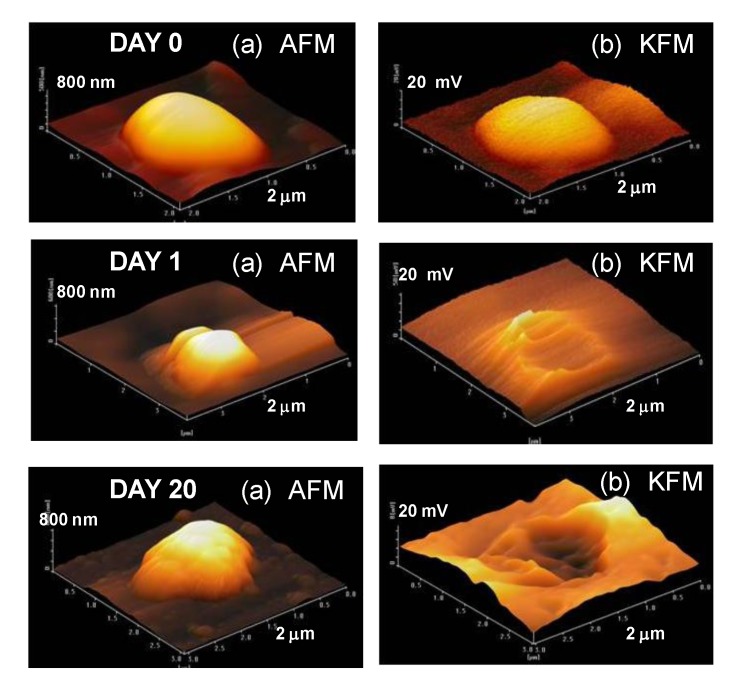
(**a**) AFM and (**b**) KFM images of uncoated Al nanoparticles subjected to corrosion test for day 0, day 1, day 20.

AFM and KFM measurements were carried out for uncoated Al nanoparticles after the corrosion test in Figure 11. The KFM image for the uncoated Al particle at day zero is represented by a half globe, almost the same as the AFM image. On the other hand, although the AFM images for uncoated Al particle at day one are half globes, the KFM images display broken shapes. For example, the top (highest) potential in KFM is changed from 18, 7, 1 mV at day 0, 1, 20, respectively. Thus, the potential value around the Al particles is thought to be very low showing the start of the electrochemical reaction by galvanic corrosion between the Al particle and the Pt plate. In the same way, the KFM image for the uncoated sample at 20 days display a broken shape. This fact suggests that the electrochemical reaction of a sample continues to 20 days. In this way, it is possible to measure the corrosion reaction of nanoparticles precisely by AFM and KFM.

**Figure 12:**
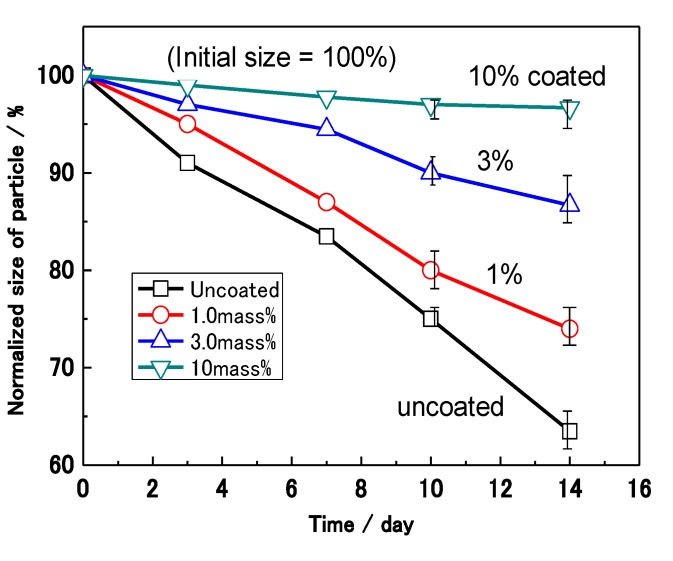
Effect of coating mass% on the sizes of Al nanoparticles caused by the wet and dry corrosion test with NaCl.

Figure 12 shows the effect of the coating mass% on the sizes of the Al nanoparticles after a wet and dry NaCl corrosion test. The size of the Al nanoparticles was normalized for the 20 samples by the supposition that the average of the initial diameter of the particles was 100%. The normalized size (S_N_) of uncoated Al decreases remarkably with time in the corrosion test, showing that the corrosion rate is very high. On the other hand, the S_N_ of the 10 mass% coated Al hardly changes. This fact suggests that corrosion is completely prevented by the polymer film in the case of the 10 mass% coated Al. After 14 days, the S_N_ of the 0, 1.0, 3.0, and 10.0 mass% coated samples are 63%, 74%, 87% and 97%, respectively. Thus, the S_N_ after 14 days shows a higher value as the coating mass% increases. It was found that the corrosion reaction is suppressed in proportion to the mass% of polymer coating.

**Figure 13:**
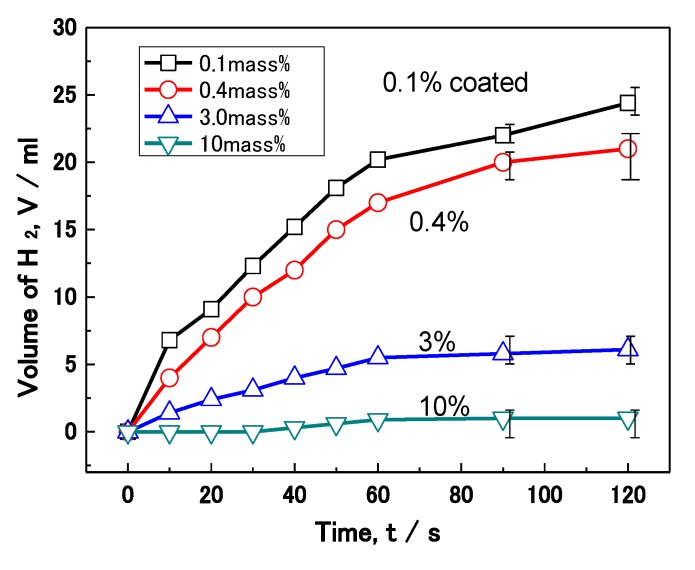
Effect of coating mass% on the hydrogen (H_2_) evolution reaction on Al nanoparticles.

Figure 13 shows the effect of polymer coating mass% on the corrosion of Al nanoparticles in an alkali solution. The volume of H_2_ evolution reaction for 70 mg of Al nanoparticles was measured in a 0.5 M NaOH solution. The volume of H_2_ evolving for each sample increases with the test time in the figure. After 120 s, the 0.1, 0.4, 3.0 and 10 mass% coated Al has an evolution volume of 24, 21, 6, and 1 mL, respectively. Thus, less H_2_ evolution is shown as the coating mass% increases, which is just the same as the previous wet and dry corrosion test in Figure 12. In this way, the H_2_ evolution reaction was also suppressed in proportion to the mass% of polymer coating.

**Figure 14:**
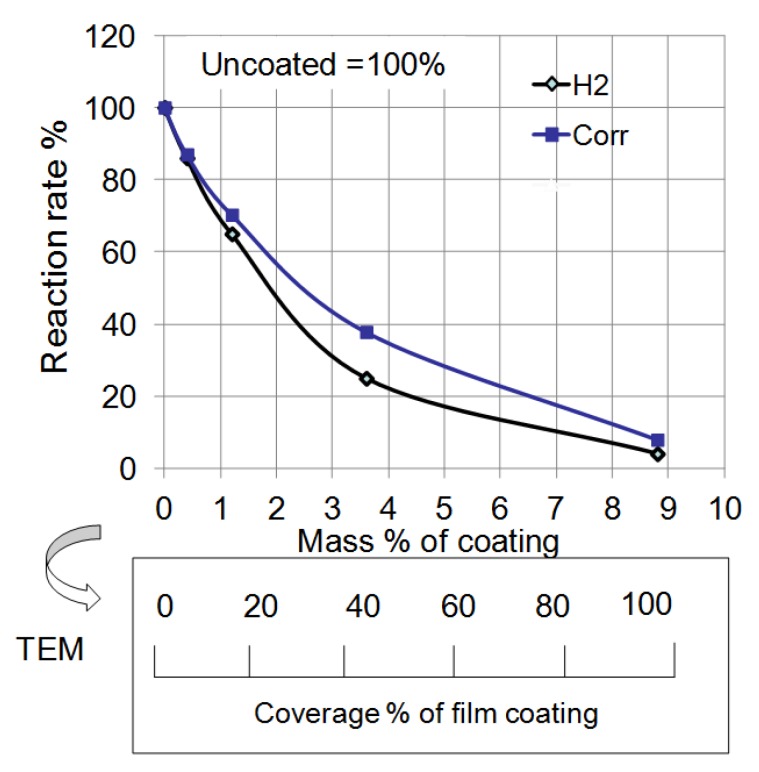
Effect of mass% of coating on the reaction rate of H_2_ evolution and corrosion from Figure 12 and Figure 13. Mass% of coating was changed to coverage percentage of film coating by TEM analysis.

Figure 14 summarizes the effect of mass% of polymer coating on the reaction rate in each experiment from Figure 12 and Figure 13. The rate of corrosion for each coated sample was determined by the supposition that the amount of corrosion of uncoated Al at 14 days was 100%. In the same way, the rate of the H_2_ reaction was determined by the supposition that the volume of uncoated Al at 120 s was 100%. Both rates were reduced as the mass% of polymer coating increased. This fact indicates that corrosion reactions on the Al particles under these conditions are suppressed by the polymer coating. Moreover, a quantitative relationship between the corrosion rate and the mass% of coating was established. The film coating coverage percentage was determined by the results of EDAX from TEM (Figure 6). It was also demonstrated that the corrosion rate of Al nanoparticles was suppressed in proportion to the coverage percentage of the polymer coating.

From these experiments, the corrosion rate of Al nanoparticles was able to be quantitatively controlled by the surface coverage percentage of the polymer coating. Moreover, almost all of the polymer synthesized was consumed in the formation of the surface film. In other words, we can control the polymer coating mass% using the mass% of polymer in the synthesis. In addition, the film thickness was almost the same—10 nm for each coating. Thus, we can control the coverage percentage of polymer coating very easily from the mass% of polymer in the synthesis. Besides, as the film thickness is very thin, each nanoparticle is able to be coated separately without bonding to one another in the synthesis. In this way, the coating of a nanometal can be established using only the mass% ratio of polymer to metal in the synthesis.

Finally, it can be said that we can prevent the corrosion of nanometals by using a polyurethane polymer coating. Thus, it is possible to store nanometals without corrosion in the atmosphere at high humidity.

## 4. Conclusions

In order to prevent corrosion, aluminum nanoparticles were coated with polyurethane polymer.

Using the EDAX analysis for F in the polymer by TEM, the coverage percentage of each coating was able to be determined. The coverage percentage of the 10 mass% and 3 mass% polymer coated Al particles in the synthesis was almost 100% and 40%, respectively. However, the film thickness was almost 10 nm for both coatings. As the film thickness was very thin, each nanoparticle was able to be coated separately without bonding to one another in the synthesis.

In AFM, the potential around the Al particles showed a lower value with increased polymer coating, indicating that the conductivity of the nano Al was isolated from the Pt plate by the polymer. The reaction rates for both wet/dry and H_2_ evolution tests were reduced as the mass% of polymer coating increased. Thus, the corrosion reaction on the Al particles was quantitatively suppressed by the polymer coating.

The corrosion rate of a nanometal could be reduced completely by using a polymer coating on the surface. Thus, it is possible to keep nanometals without corrosion in the atmosphere at high humidity.
